# Phenolic Compounds and Bioactivity of *Cytisus villosus* Pourr.

**DOI:** 10.3390/molecules23081994

**Published:** 2018-08-10

**Authors:** Amel Bouziane, Boulanouar Bakchiche, Maria Inês Dias, Lillian Barros, Isabel C.F.R. Ferreira, Husam A. AlSalamat, Sanaa K. Bardaweel

**Affiliations:** 1 Laboratory of Process Engineering, Faculty of Technology, Laghouat University, BP 37 G Ghardaia Road, 03000 Laghouat, Algeria; a.bouziane@mail.lagh-univ.dz (A.B.); b.bakchiche@lagh-univ.dz (B.B.); 2 Centro de Investigação de Montanha (CIMO), Instituto Politécnico de Bragança, Campus de Santa Apolónia, 5300-253 Bragança, Portugal; maria.ines@ipb.pt (M.I.D.); lillian@ipb.pt (L.B.); 3 Department of Pharmaceutical Sciences, School of Pharmacy, University of Jordan, Amman 11942, Jordan; husam.salamat@gmail.com

**Keywords:** *Cytisus villosus*, phytochemical, antioxidant, antimicrobial, antiproliferative activity

## Abstract

The present study focuses on the chemical composition, antioxidant, antimicrobial, and antiproliferative activities of the ethyl acetate and aqueous extracts obtained from the aerial parts of *Cytisus villosus* Pourr. HPLC-DAD-ESI/MSn was used to identify the phenolic compounds, being (epi)gallocatechin dimer the major compound (111 ± 5 µg/g·dw) in the aqueous extract, while myricetin-*O*-rhamnoside (226 ± 9 µg/g·dw) was the main molecule in the ethyl acetate extract. Both extracts exhibited good scavenging activities against DPPH radical (EC_50_ µg/mL of 59 ± 2 and 31 ± 2 for aqueous and ethyl acetate extracts, respectively). However, the ethyl acetate extract demonstrated more potent quenching activities than the aqueous extract. The antimicrobial activities were assessed on selected Gram-positive (*Staphylococcus epidermidis*) and Gram-negative (*Escherichia coli* and *Pseudomonas aeruginosa*) bacteria, as well as on pathogenic fungus *Candida glabrata*. The extracts possessed selective and potent antimicrobial activities against the Gram-positive bacterium (IC_50_ of 186 ± 9 μg/mL and 92 ± 3 μg/mL for aqueous and ethyl acetate extracts, respectively). Finally, *C. villosus* extracts were evaluated for their antiproliferative potential on three human cancer cell lines representing breast and colon cancers. Although both extracts demonstrated sufficient growth inhibition of the three different cell lines, the ethyl acetate extract exhibited higher activity (LD_50_ values of 1.57 ± 0.06 mg/mL, 2.2 ± 0.1 mg/mL, and 3.2 ± 0.2 mg/mL for T47D, MCF-7, and HCT-116 cell lines). Both the extracts obtained from the aerial parts of *C. villosus* revealed very promising results and could be applied as functional agents in the food, pharmaceutical, and cosmeceutical industries.

## 1. Introduction

The genus *Cytisus* (*Fabaceae* family) is prevalent in the Mediterranean Basin, encompassing about 70 species, eight of which grow in Northern Algeria. This genus has been reported, in folk medicine, as a diuretic and for the treatment of mild hypertension [1]. Additionally, a leaf decoction has also been reported to be effective in relieving respiratory tract problems. Several species of the genus *Cytisus* have been used in traditional medicine, mainly for their antioxidant, cytoprotective, diuretic, hypnotic, anxiolytic, antiparasitic and antidiabetic potentials [1,2,3,4]. The most commonly known *Cytisus scoparius* (L.) link, a widely used traditional Chinese herb, is taken for nourish Yin and to invigorate the heart and liver [1]. It is also well known as a stimulating cardiac tonic and diuretic and has been reported as useful remedy in managing heart failure and cardiac edema [5]. In addition, *Cytisus multiflorus *(L’Hér*.)* Sweet (Spanish broom) is frequently used in folk medicine, and it is reported to have various health benefits, including anti-inflammatory properties [6].

Interestingly, *Cytisus villosus* Pourr., also known as hairy broom, is distributed in Northern Africa, occurring from the mountains of Central and Northern Morocco to the mountains of Cape Bon in Tunisia [7]. The plant height ranges between 50–200 cm with wintering buds that grow at a height between 12 and 20 cm (maximum 50 cm); in cold seasons the herbaceous portions dry up and only the woody and hypogeal parts remain alive. Although the traditional use of this plant has been reported for controlling hypertension [8], no previous reports on the phytochemical composition or the evaluation of its biological activities can be found in literature. Therefore, the present study aimed to study the ethyl acetate and aqueous extracts obtained from the aerial parts *Cytisus villosus* Pourr., in terms of its phytochemical composition, more precisely the phenolic compounds profile obtained by HPLC-DAD-ESI/MSn, and evaluate other potential bioactivities of this plant, such as antioxidant, antimicrobial, and antiproliferative activities.

## 2. Results

### 2.1. Phytochemical Characterization of C. villosus Aqueous and Ethyl Acetate Extracts

The yield of extractable components relative to the weight of dried plant material ranged from 21.25% for aqueous extract and 5.85% for ethyl acetate extract. To the best of the authors’ knowledge, there are no reports on the phytochemical composition of *C. villosus*, being therefore a novel finding and of an extreme importance for the prospection of plants with biomolecules of high added value and with bioactive potential.

**Figure 1:**
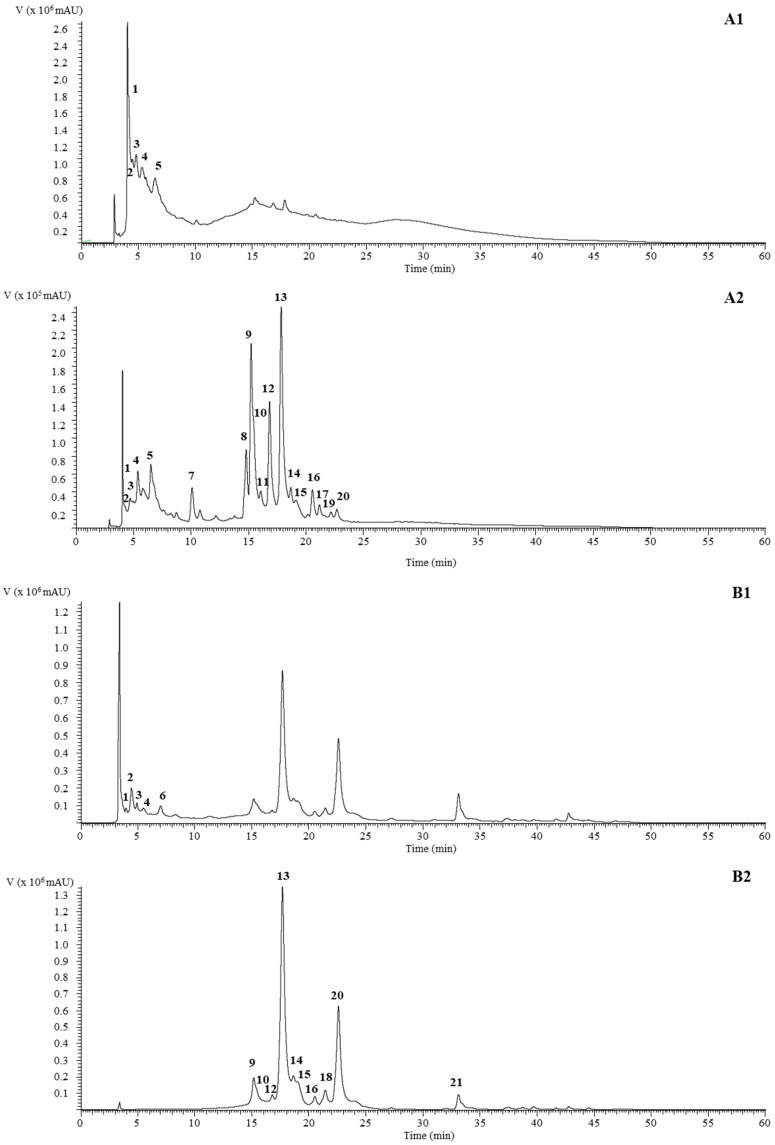
Phenolic profile of aqueous and ethyl acetate extract of *C. villosus* recorded at 280 nm (**A1** and **B1**, respectively) and 370 nm (**A2** and **B2**, respectively).

The peak characteristics, tentative identification and quantification of phenolic compounds present in aqueous and ethyl acetate extracts of *C. villosus* are presented in Table 1. An exemplary phenolic profile of the both extracts, recorded at 280 and 370 nm, is shown in Figure 1. Twenty-one different phenolic compounds were found in *C. villosus* extracts divided in three main families: flavan-3-ols (catechin derivatives), flavonols (myricetin, quercetin, kaempferol glycoside derivatives) and flavones (apigenin glycoside derivatives).

Myricetin derivatives were the major type of flavonols found in *C. villosus* extracts, being identified eight compounds which represented 20% of the total polyphenols found in aqueous extracts and 47% in the ethyl acetate extract. Peak **9** was positively identified as myricetin-3-*O*-glucoside according to its retention time, mass and UV-vis characteristics by comparison with commercial standards and bibliographic reference with other Fabaceae samples, cowpeas [9]. Peaks **8** and **10** were tentatively identified as myricetin-3-*O*-rutinoside ([M − H]^−^ *m*/*z* 625) and myricetin-*O*-hexoside ([M − H]^−^ *m*/*z* 479), respectively, by comparison with bibliographic descriptions in *Melicoccus bijugatus* Jacq.‘Montgomery’fruits [10] and *C. incanus* leaves [11], respectively. Peaks **11/12** ([M − H]^−^ *m*/*z* 565) and **13/15** ([M − H]^−^ *m*/*z* 463) were tentatively identified as myricetin-*O*-malonylhexoside and myricetin-*O*-deoxyhexoside by comparison with bibliographic descriptions in *Cichoriumintybus* [12] and *C. incanus* leaves [11], respectively.

Finally, peak **19** presented a pseudomolecular ion at [M − H]^−^ *m*/*z* 771, and subsequent fragmentation pattern at *m*/*z* 625 (146 mu) and *m*/*z* 317 (308 mu), corresponding to the loss of a coumaroyl (due to the late retention time is considered the loss of an acid molecule) and rutinosyl moieties, respectively, being tentatively identified as myricetin-*O*-coumaroylrutinoside.

The second major family of flavonoids found in *C. villosus* extracts were quercetin derivatives. Peak **14** was positively identified as quercetin-3-*O*-glucosidein comparison with the commercial standard. Peak **16** ([M − H]^−^ *m*/*z* 549) was tentatively identified as quercetin-*O*-malonylhexoside, by comparison with bibliographic descriptions of this compound in *Cichorium intybus* [12]. Peaks **18** and **20** were tentatively identified as quercetin-*O*-pentoside ([M − H]^−^ *m*/*z* 433) and quercetin-*O*-rhamnoside ([M − H]^−^ *m*/*z* 447), respectively, by comparison with literature descriptions of these compounds in *C. incanus* leaves [11]. Two kaempferol derivatives were found, peaks **17** and **21**.

Furthermore, Peak **17** was positively identified as kaempferol-3-*O*-rutinoside by comparison with the commercial standard. Peak **21** was tentatively identified as kaempferol-*O*-coumaroylhexoside (commonly identified as tiliroside), presenting a pseudomolecular ion at [M − H]^−^ *m*/*z* 593, a late retention time and low UV-vis spectra, indicates the presence of an acid molecule in the structure, as previously described by Jabeur et al. [17] in *Tilia platyphyllos* extract. Finally, only one flavone, a apigenin derivative, was found in *C. villosus* aqueous extract, demonstrating to be a *C*-glycoside due to the characteristic loss of 90 and 30 mu moieties, being tentatively identified as apigenin-*C*-hexoside-*C*-hexoside (peak **7**, [M − H]^−^ *m*/*z* 593), as previously described by Ferreres et al. [16]; nonetheless, it was not possible to number the *C*-glycoside residue positions due to the lack of a standard compound for comparison.

Regarding flavan-3-ols family, six compounds were identified. Peak **6** was positively identified as (+)-catechin by comparison with a commercial standard and also using bibliographic reference with other Fabaceae samples, such as common bean [15]. The majority of flavan-3-ol compounds found in the samples were tentatively identified as (epi) gallocatechin isomer (peaks **2**, **3**, and **4**, [M − H]^−^ *m*/*z* 305) and (epi) gallocatechin dimer (peak **1**, [M − H]^−^ *m*/*z* 609), which were identified by comparison with the compounds found in *Parkia biglobosa* (Jacq.) G. Don. belonging to Fabaceae family [13]. Galloyl-HHDP-glucoside (peak **5**, [M − H]^−^ *m*/*z* 633), an hydrolizable tannin, was also tentatively identified in *C. villosus* extracts by comparison with bibliographic description of this compound by Kim et al. [14].

The total phenolic compounds found in both extracts, aqueous and ethyl acetate, was very similar, 712 and 697 µg/g dry weight, respectively. However, the differences were found in total flavan-3-ols and flavonols/flavone families, where the aqueous extract showed a higher amount of flavan-3-ols and flavones and the ethyl acetate extract presented higher flavonols with the absent of flavones (496 and 550 µg/g dry weight, respectively). (Epi)gallocatechin dimer (peak **1**) was the major compound found in aqueous extract, while myricetin-*O*-rhamnoside (peak **14**) was the main compoundpresent in the ethyl acetate extract.

### 2.2. Antioxidant Activity

The antioxidant activity of each extract was determined by DPPH and ABTS methods. The free radical scavenging activity determined by DPPH was expressed as the EC_50_ value (the effective concentration of extract required to inhibit 50% of the initial DPPH free radical). A low value of EC_50_ indicates higher antioxidant activity and results are shown in Table 2. A higher free radical scavenging activity was obtained with the ethyl acetate extract (EC_50_ = 31 µg/mL), while the aqueous extract showed a higher value, with an EC_50_ value of 59 µg/mL. Both extracts revealed lower activity compared to ascorbic acid (control standard, EC_50_ value of 3.1 µg/mL). Indeed, due to different antioxidant potentials of different compounds, the antioxidant activity is strongly dependent on the type of extraction performed. Ait-Kaci Aourahoun et al. [18] studied the hydroalcoholic extracts of leaves and stems of *C. villosus*, with EC_50_ = 19.17 and 77.81 µg/mL, respectively, for the DPPH radical scavenging activity, these results are comparable to the herein obtained results.

The scavenging activity of *C. villosus* extracts using the ABTS radical is presented in Table 2. The data indicated that both aqueous and ethyl acetate extracts were effective scavenger of ABTS radical and this activity was comparable with ascorbic acid. The ethyl acetate extract of *C. villosus* exhibited stronger antioxidant activity than the aqueous extract, revealing EC_50_ values of 232 and 468 µg/mL, respectively.

The differences observed between the two extracts are probably due to the dissimilar contents of the identified phenolic compounds present in both extracts. As observed from the results, ethyl acetate extracts exhibited a stronger activity than the aqueous extract. Thus, it may be concluded that the ethyl acetate extract contains more bioactive compounds than the aqueous extract, and could be relatively stronger scavengers of free radicals.

### 2.3. Antimicrobial Activity

The aqueous and ethyl acetate extracts were studied regarding their potential antimicrobial activity against a panel of pathogenic microorganisms, including Gram-positive and Gram-negative bacteria, and fungus (Table 2). There was a selective antimicrobial activity against the Gram-positive bacterium used in this study, *Staphylococcus epidermidis*, observing IC_50_ values of 189 and 92 μg/mL for the aqueous and ethyl acetate extracts, respectively. However, poor activities were demonstrated when the extracts were tested against Gram-negative bacteria *Escherichia coli* and *Pseudomonas aeruginosa.* On the other hand, moderate antifungal activity was associated with both extracts when applied to *Candida glabrata* (IC_50_ values of 467 and 226 µg/mL for the aqueous and ethyl acetate extracts, respectively).

Interestingly, the ethyl acetate extract was more potent than the aqueous extract, as shown in Table 2, suggesting a better penetration to the cellular compartment or an enhanced ability to complex with bacteria cell wall and therefore, inhibiting the microbial growth. Several literature reports support the antimicrobial potential of multiple constituents of *C. villosus* extracts, for example, myricetin has been reported to be a very effective phenolic compound in the inhibition of some species of Gram-positive and Gram-negative pathogenic bacteria [19,20]. Similarly, quercetin extracted from different plants was evaluated against several pathogenic bacteria, including Gram-positive and Gram-negative species, be reported to be a highly efficient antimicrobial agent against most of the examined bacterial strains tested in vitro [19,20,21].

### 2.4. Antiproliferative Activity

**Figure 2:**
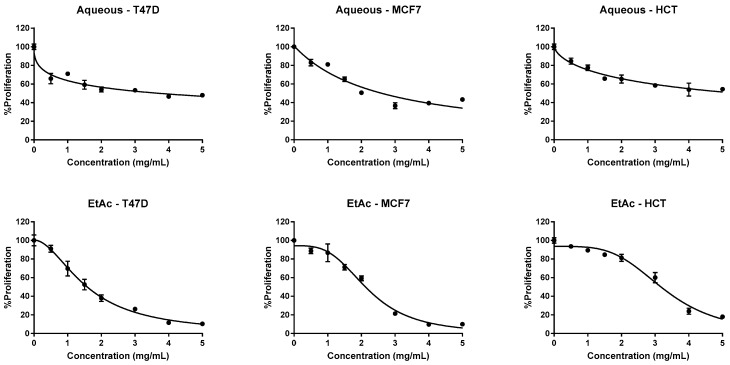
Antiproliferative activities of *C. villosus* extracts against three human cancer cell lines. Exposure time 48 h. Values are expressed as mean ± SD of three experiments.

To the best of our knowledge, the antiproliferative activities associated with *C. villosus* are being reported for the first time in this manuscript. It was observed that both extracts inhibited the growth of human cancer cell lines, with higher activity for the ethyl acetate fraction, and LD_50_ values ranging between 1.57–3.2 mg/mL and 2.6–5.4 mg/mL for the ethyl acetate and aqueous extracts, respectively (Table 2, Figure 2). The demonstrated compelling antiproliferative activities may be attributed to the distinctive composition of the prepared extracts of *C. villosus*.

The phytochemical composition of both the aqueous and ethyl acetate extracts appear to be rich in some of the most potent naturally occurring antiproliferative phytochemicals, such as myricetin, quercetin, epigallocatechin, and kaempferol [22,23,24,25]. For instance, kaempferol has been reported to have strong antioxidant and antiproliferative activities and also decreased the expression of vascular endothelial growth factor (VEGF), a marker of angiogenesis, in several types of cancer cells [26]. Similarly, polyphenols, such as quercetin, are frequently reported to have antineoplastic properties against numerous cancers from different origins [25]. Interestingly, the multiple phytochemical components of the extracts and the effectiveness of the extracts to suppress human cancer cells growth at relatively low concentrations may suggest that the observed activities are rather due to additive or synergistic effects of the individual constituents.

## 3. Materials and Methods

### 3.1. Samples and Extract Preparation

The aerial parts of *C. villsous* were collected in April 2017, from the Aflou region of Laghouat city (located in the south part of the Algerian Saharan Atlas). The plant was identified by the botanist Dr. Mohamed Kouidri, (Department of Agronomy, Faculty of Sciences, University of Laghouat-Algeria) and voucher specimens (LGP Cv/04/17) were deposited at the Laboratory of Process Engineering, University of Laghouat.

The aerial parts of *C. villosus* plant were dried at room temperature and grounded to powder (40 mesh size). This powder was extracted by maceration using a mixture of ethanol/water (80:20, *v*/*v*) for 24 h, at a solid/liquid ration of 5% (*w*/*v*), with continuous stirring. Afterward, a filtration step using a Büchner funnel under reduced pressure was performed and any residual of ethanol was removed under vacuum in a rotary evaporator at 40 °C. The remaining solution was defatted twice with hexane to remove lipids as previously described [27], and the aqueous phase was collected while the organic phase was discarded. Afterwards, the obtained solution was extracted with equal volume of ethyl acetate. The upper phase of ethyl acetate fraction was then collected and dried by adding a sufficient amount of anhydrous sodium sulphate and then evaporated to dryness using a rotary evaporator. The dried precipitate was then dissolved in 10 mL of ethanol and kept at 4 °C, until further assays. At the end of this procedure, two phenolic extracts (upper phase—ethyl acetate extract and the lower phase−water−extract) were obtained from the aerial parts of *C. villosus*.

### 3.2. Characterization of Phenolic Compounds by HPLC-DAD-ESI/MSn

Chromatographic analysis was performed in a Dionex Ultimate 3000 UPLC (Thermo Scientific, San Jose, CA, USA) system equipped with a diode array detector (280, 330 and 370 nm as preferred wavelengths), coupled to a mass spectrometer (linear Ion Trap LTQ XL mass spectrometer, Thermo Finnigan, San Jose, CA, USA) equipped with an ESI source and working in negative mode [28]. The phenolic compounds were identified by comparing their retention times, UV and mass spectra with those obtained from standard compounds. Otherwise, compounds were tentatively identified comparing the obtained information with available data reported in the literature. For quantitative analysis, a calibration curve for each available phenolic standard was constructed based on the UV signal. For the identified phenolic compounds for which a commercial standard was not available, the quantification was performed through the calibration curve of the most similar available standard and results were expressed in µg per g of dry weight (µg/g·dw).

### 3.3. Antioxidant Activities

#### 3.3.1. Scavenging Activity of DPPH Radical

DPPH radical scavenging activity was determined according to Boulanouar et al. [29] with some modifications. In this study, 0.1 mL of each extract (at different concentrations, ranging from 100 to 10 µg/mL) was mixed with 1.9 mL of 60 µM DPPH methanol solution. The reaction was allowed to stand at room temperature in the dark for 30 min and then the absorbance was recorded at 517 nm. The scavenging activity was estimated using the following equation: Scavenging effect (%) = [100 × (A_C_ − A_S_/Ac)], where Ac is the absorbance of the control reaction (containing all reagents except the test sample) and A_S_ the absorbance of the tested sample. The concentration of each extract that could scavenge 50% of the DPPH radicals (EC_50_) was calculated. Ascorbic acid was used as positive reference.

#### 3.3.2. Scavenging Activity of ABTS Radical

ABTS radical scavenging activity was determined with a method previously described [30] with some modifications. ABTS radical cation (ABTS^•+^) was produced by reacting 7 mM ABTS stock solution with 2.45 mM potassium persulphate and allowing the mixture to stand in the dark at room temperature for 12–16 h. The ABTS^•+^ solution (stable for 2 days) was diluted with methanol to an absorbance of 0.70 ± 0.02 at 734 nm. After addition of 20 µL of sample (at different concentrations, ranging from 500 to 100 µg/mL) or ascorbic acid (control standard) to 1980 µL of diluted ABTS^•+^ solution, it was left to stand in dark at room temperature for 6 min, and the absorbance was measured at 734 nm. For each extract, the percentage inhibition of ABTS^•+^ was calculated according to the equation: (%) = [100 × (A_C_ − A_S_/Ac)], where Ac is the absorbance of the control reaction (the absorbance of the ABTS solution) and A_S_ the absorbance of the tested sample. The concentration of extract that could scavenge 50% of the ABTS radicals (EC_50_) was calculated.

### 3.4. Antimicrobial Activity

#### 3.4.1. Microorganisms

Three bacterial and one fungal species were obtained from the Microbial Culture Collection Center of Medicine School at The University of Jordan, Amman, Jordan. The species used were: *Staphylococcus epidermidis* ATCC 12228 (Gram-positive bacterium), *Escherichia coli* ATCC 29425 and *Pseudomonas aeruginosa* ATCC 15442 (Gram-negative bacteria) and *Candida glabrata* ATCC 22553 (fungus). The three microorganisms represent important food pathogens that are commonly encountered.

#### 3.4.2. Determination of Median Inhibitory Concentration (IC_50_)

The antimicrobial activity of the prepared aqueous and ethyl acetate extracts (ranging concentrations: 2000 to 50 µg/mL) was determined by the median inhibitory concentration (IC_50_); defined as the concentration at which microorganism survival is reduced by 50%. The percentage (%) of the microbial growth inhibition was calculated based on the following equation: [(Ac − At)/Ac] × 100, where Ac was an average of three triplicates of OD_600_ values of the positive growth controls, non-treated, and At was an average of three triplicates of OD_600_ values of the cultures treated with either extract. The IC_50_ was determined using linear relation between the percent of growth inhibition and concentration and expressed as the mean ± standard deviation of three independent experiments [31]. Concisely, to achieve a final inoculum size of 1 × 10^5^ CFU/mL, microorganisms were cultured in 96 flat bottom microtiter plates (TPP, Trasadingen, Switzerland), where each well was filled with 100 μL nutrient broth inoculated with 10 μL aliquot of the microorganism. Positive controls, Ampicillin for bacteria and Amphotericin B for fungi, and a negative control of the vehicle (DMSO), were prepared under the same experimental conditions and employed in triplicates. Bacterial plates were incubated for 24 h at 37 °C, whereas the fungi plates were incubated for 48 h at 33 °C, with shaking. At the end of the incubation time, optical densities were measured at 600 nm (OD_600_) using a microplate reader (Tri-Bio Science, Palo Alto, CA, USA).

### 3.5. Antiproliferative Activity

#### 3.5.1. Cell Lines and Cell Viability

The cancer cell lines (MCF7, T47D and HCT116) were purchased from the American Type Culture Collection (Rockville, MD, USA). All cells were maintained in culture medium DMEM (Dulbecco’s Modified Eagle’s Medium), supplemented with 10% fetal bovine serum, 100 U/mL of penicillin, 100 µg/mL of streptomycin, at 37 °C with 5% of CO_2_. The number of living cells was evaluated by the Trypan blue method [32].

#### 3.5.2. MTT Assay

The antiproliferative activity of the aqueous and ethyl acetate extracts were evaluated in 96-well round bottomed microplates using the MTT assays (3-[4,5-dimethylthiazole-2-yl]-2,5-diphenyl-tetrazolium bromide) (Sigma-Aldrich, St. Louis, MO, USA) [33]. Cells were cultured in the 96 well plates at cell density of 1 × 10^4^ cells/mL and incubated for 24 h to allow adherence. Subsequently, extracts were added to each well at the concentration range of 0.5 to 10 mg/mL and incubated for 48 h. At the end of exposure time, 20 µL of 0.5 mg/mL of MTT was added to each well and incubated for 4 h, afterward its reduction to formazan within viable cells was calculated by measuring the absorbance at 570 nm. The percent of growth inhibition was calculated using the following equation: inhibition (%) = 100 − (mean of Abs of test sample-mean of Abs of negative control) × 100/(mean of Abs of positive control-mean of Abs of negative control). The GraphPad Prism 7 software (GraphPad Software, San Diego, CA, USA) was used for data analysis to calculate inhibition percentage and results were expressed as LD_50_ value, defined as the concentration causing 50% growth inhibition. Doxorubicin was used as a positive control and applied to wells in the same manner as the studied extracts.

### 3.6. Statistical Analysis

For all the extracts and tests, the analyses were carried out in triplicate and the obtained values were expressed as the mean ± standard deviation (SD). The results were analysed using a Student’s *t*-test, with α = 0.05. The analyses were carried out using IBM SPSS Statistics for Windows, Version 22.0 (IBM Corp., Armonk, New York, NY, USA).

## 4. Conclusions

Overall, this study reported a full characterization of the chemical and biological profiles of *C. villosus* ethyl acetate and aqueous extracts. The chemical composition of both extracts revealed that the total phenolic compounds were very similar. However, the aqueous extract presented higher amounts of flavan-3-ols, mainly due to (epi)gallocatechin dimer, and the ethyl acetate extract presented higher flavonols content, mainly due to myricetin-*O*-rhamnoside. The antioxidant properties of the ethyl acetate fraction appeared to be comparable to ascorbic acid in DPPH and ABTS assays and superior to those of the aqueous fraction. Selective and high antimicrobial activities were observed against the Gram-positive bacterium *Staphylococcus epidermidis.* The observed growth inhibition of breast and colon cancer cell lines could suggest a potential use for *C. villosus* extracts as a nutraceutical for cancer prevention. Thus, both these extracts revealed to have promising bioactivities, that could be correlated to the different phenolic content in each extract. Thus, being interesting to be explored as a functional ingredient in the food, pharmaceutical, and cosmetic area.

## Figures and Tables

**Table 1:** Retention time (Rt), wavelengths of maximum absorption in the visible region (λ_max_), mass spectral data, identification and quantification of phenolic compounds in *C. villosus* extracts (mean ± SD).

Peak	Rt (min)	λmax (nm)	[M − H]^−^ (*m*/*z*)	MS^2^ (*m*/*z*)	Tentative Identification	Quantification (µg/g·dw)			Reference Used for Identification
						Aqueous Extract	Ethyl Acetate Extract	Student’s *t*-Test	
1	4.0	271	609	483 (14), 441 (100), 423 (28), 305 (11)	(Epi)gallocatechindimer ^(1)^	156 ± 2	7.8 ± 0.2	<0.001	Tala et al. [13]
2	4.4	272	305	219 (78), 179 (100), 125 (12)	(Epi)gallocatechin isomer 1 ^(1)^	70 ± 3	59 ± 2	<0.001	Tala et al. [13]
3	4.9	271	305	219 (77), 179 (100), 125 (15)	(Epi)gallocatechin isomer 2 ^(1)^	111 ± 5	20.35 ± 0.08	<0.001	Tala et al. [13]
4	5.4	271	305	219 (70), 179 (100), 125 (20)	(Epi)gallocatechin isomer 3 ^(1)^	85 ± 2	23.3 ± 0.4	<0.001	Tala et al. [13]
5	6.5	273	633	467 (23), 301 (100)	Galloyl-HHDP-glucoside ^(2)^	74 ± 2	nd	-	Kim et al. [14]
6	7.0	278	289	245 (100), 205 (35), 137 (5)	(+)-Catechin ^(1)^	nd	36.1 ± 0.7	-	DAD/MS; Chen et al. [15]
7	10.1	330	593	503 (28), 473 (100), 383 (10), 353 (19), 311 (8)	Apigenin-*C*-hexoside-*C*-hexoside ^(3)^	13.7 ± 0.4	nd	-	Ferreres et al. [16]
8	14.8	353	625	317 (100)	Myricetin-3-*O*-rutinoside ^(4)^	8.7 ± 0.4	nd	-	Bystrom et al. [10]
9	15.2	356	479	317 (100)	Myricetin-3-*O*-glucoside ^(4)^	25.0 ± 0.8	32.1 ± 0.2	<0.001	DAD/MS; Nderitu et al. [9]
10	15.5	356	479	317 (100)	Myricetin-*O*-hexoside ^(4)^	18.7 ± 0.5	19.1 ± 0.3	0.215	Gori et al. [11]
11	16.0	354	565	521 (100), 479 (22), 317 (57)	Myricetin-*O*-malonylhexoside ^(4)^	13.0 ± 0.2	nd	-	Carazzone et al. [12]
12	16.8	356	565	521 (100), 479 (16), 317 (63)	Myricetin-*O*-malonylhexoside ^(4)^	24.2 ± 0.9	20.99 ± 0.08	0.001	Carazzone et al. [12]
13	17.8	351	463	317 (100)	Myricetin-*O*-rhamnoside ^(4)^	38.1 ± 0.5	226 ± 9	<0.001	Gori et al. [11]
14	18.7	352	463	301 (100)	Quercetin-3-*O*-glucoside ^(4)^	13.5 ± 0.5	34.9 ± 1	<0.001	DAD/MS
15	19.2	351	463	317 (100)	Myricetin-*O*-deoxyhexoside ^(4)^	13.3 ± 0.3	30.4 ± 0.6	<0.001	Gori et al. [11]
16	20.6	353	549	505 (100), 463 (15), 301 (44)	Quercetin-*O*-malonylhexoside ^(4)^	13.7 ± 0.2	20.7 ± 0.4	<0.001	Carazzone et al. [12]
17	21.2	340	593	285 (100)	Kaempferol-3-*O*-rutinoside ^(4)^	11.8 ± 0.2	nd	-	DAD/MS
18	21.5	354	433	301 (100)	Quercetin-*O*-pentoside ^(4)^	nd	26.3 ± 0.8	-	Gori et al. [11]
19	22.2	-	771	625 (100), 317 (15)	Myricetin-*O*-coumaroylrutinoside ^(4)^	11.11 ± 0.09	nd	-	DAD/MS
20	22.7	350	447	301 (100)	Quercetin-*O*-rhamnoside ^(4)^	11.7 ± 0.1	111 ± 2	<0.001	Gori et al. [11]
21	33.1	314	593	447(18), 285(100)	Kaempferol-*O*-coumaroylhexoside ^(4)^	nd	27.4 ± 0.5	-	Jabeur et al. [17]
					Total flavan-3-ols	496 ± 1	147 ± 2	<0.001	-
					Total flavonols	203.3 ± 4.1	550 ± 12	<0.001	-
					Total flavones	13.7 ± 0.4	nd	-	-
					Total phenolic compounds	712 ± 3	697 ± 10	0.019	-

nd—Not detected (below LOD). dw—dry weight. Standard calibration curves: ^(1)^ catechin (*y* = 84950*x* − 23200; *R*^2^ = 0.999); ^(2)^ ellagic acid (*y* = 26719*x* − 317255; *R*^2^ = 0.999); ^(3)^ apigenin-6-*C*-glucoside (*y* = 107025*x* + 61531; *R*^2^ = 0.999); ^(4)^ quercetin-3-*O*-glucoside (*y* = 34843*x* − 160173; *R*^2^ = 0.999). A Student’s *t*-test was used to determine the significant difference between two different samples, with α = 0.05.

**Table 2:** Antioxidant, antimicrobial and antiproliferative activities of *C. villosus* extracts (mean ± SD).

Extract	Antioxidant Activity ^a^		Antimicrobial Activity ^b^				Antiproliferative Activity ^c^		
	DPPH Scavenging Activity	ABTS Scavenging Activity	*Staphylococcus epidermidis*	*Escherichia coli*	*Pseudomonas aeruginosa*	*Candida glabrata*	T47D	MCF-7	HCT-116
**Aqueous**	59 ± 2	468 ± 34	186 ± 9	1029 ± 32	1158 ± 12	467 ± 13	3.8 ± 0.2	2.6 ± 0.1	5.4 ± 0.1
**Ethyl acetate**	31 ± 2	232 ± 2	92 ± 3	1149 ± 28	887 ± 10	226 ± 9	1.57 ± 0.06	2.2 ±0.1	3.2 ± 0.2
**Student’s *t*-test**	<0.001	<0.001	<0.001	<0.001	<0.001	<0.001	<0.001	<0.001	<0.001

**^a^** Antioxidant activity of *C. villosus* extracts. The antioxidant activity was expressed as EC_50_ values (EC_50_ µg/mL, mean ± SD), which means that higher values correspond to lower antioxidant potential. EC_50_ values correspond to the sample concentration achieving 50% of antioxidant activity. Ascorbic acid EC_50_ values: 3.1± 0.1 μg/mL (DDPH) and 101 ± 3 μg/mL (ABTS scavenging). **^b^** Antimicrobial activity of *C. villosus* extracts measured by IC_50_ (μg/mL, mean ± SD). Results represent the means of three independent readings ±SD. Positive control Ampicillin IC_50_ values: 22 and 56 µg/mL for *Staphylococcus epidermidis* and *Escherichia coli* and *Pseudomonas aeruginosa*, respectively. Amphotericin B IC_50_: 14 µg/mL. **^c^** Antiproliferative activity evaluation of *C. villosus* extracts by MTT assay in the examined human cancer cell lines, exposure time 48 h. The presented LD_50_ values are expressed as mg/mL ± SD and correspond to the means of three independent readings. Doxorubicin LD_50_ values: 5–25 μg/mL. A Student’s *t*-test was used to determine the significant difference between two different samples, with α = 0.05.
